# The Three Steps Needed to End the COVID-19 Pandemic: Bold Public Health Leadership, Rapid Innovations, and Courageous Political Will

**DOI:** 10.2196/19043

**Published:** 2020-04-06

**Authors:** Jodie L Guest, Carlos del Rio, Travis Sanchez

**Affiliations:** 1 Rollins School of Public Health at Emory University Atlanta, GA United States; 2 Emory University School of Medicine Atlanta, GA United States; 3 JMIR Public Health and Surveillance JMIR Publications Atlanta, GA United States

**Keywords:** COVID-19, coronavirus, SARS-CoV-2

## Abstract

The world is experiencing the expansive spread of severe acute respiratory syndrome-coronavirus 2 (SARS-CoV-2) in a global pandemic that is placing strain on health care, economic, and social systems. Commitment to implementing proven public health strategies will require bold public health leadership and courageous acts by politicians. Developing new innovative communication, mitigation, and health care approaches, particularly in the era of social media, is also clearly warranted. We believe that the best public health evidence must inform activities in three priority areas to stop this pandemic: (1) coordinated and consistent stay-at-home orders across multiple jurisdictions, including potential nationwide mandates; (2) rapid scale-up of SARS-CoV-2 testing; and (3) improved health care capacity to respond. This editorial outlines those areas, the rationale behind them, and the call for innovation and engagement of bold public health leadership to empower courageous political action to reduce the number of deaths during this pandemic.

## Introduction

The world is experiencing the expansive spread of severe acute respiratory syndrome-coronavirus 2 (SARS-CoV-2) in a global pandemic that was first reported on December 31, 2019, in Wuhan, China. What began as cases of pneumonia with unknown etiology was identified as a novel coronavirus on January 7, 2020. Coronavirus disease 2019 (COVID-19), the illness that comes from SARS-CoV-2, was named a pandemic by the World Health Organization (WHO) on March 11, 2020 [1]. By that time, it had ravaged much of China. The epidemic is accelerating; the time from the first reported case to the first 100,000 cases was 67 days. It took 11 days for the next 100,000 cases, 4 days for the following 100,000 cases, and 3 days for the subsequent 100,000 cases. As of March 31, 2020, there are 858,669 cases and 42,151 deaths in the world attributed directly to COVID-19 (188,530 cases and 3,889 deaths in the United States, a 50.3% and 68.5% increase, respectively, in 2 days). We should remember that 1 month ago, on March 1, there were only 30 cases in the United States [2]. Every region of the globe is currently impacted by COVID-19 [3].

The enormous strain this pandemic is placing on health care systems across the world is palpable, from testing capacity to supply chains for personal protective equipment (PPE); specimen collection swabs; and supplies and equipment, including ventilators, for those requiring hospital care. New approaches are needed to scale up testing for COVID-19, to reduce the needs for PPE and specimen collection swabs and to allow testing for SARS-CoV2 outside of health care facilities.

New cases of COVID-19 infection and casualties continue to multiply and mixed messages abound. As the public health world has urgently recommended COVID-19 prevention measures, they are being questioned as being too vast, hard to follow, invasive to our lifestyle, and damaging to the economy. Public health experts have either been sidelined in the COVID-19 response decisions or have found themselves at odds with much of the information being presented by political leadership. In the United States, the country currently with the largest number of COVID-19 cases, President Trump extended the initial 15-day national slow down and called for social distancing until April 30, 2020, in an attempt to reduce the spread of the virus [4,5]. States like California, Illinois, and New York have implemented state-wide “stay-at-home” ordinances, while other states have implemented less restrictive measures or no statewide measures at all [6-8]. Determining the proper scale and timing of these measures is critical to controlling the spread of COVID-19 and the numbers of lives lost. We believe that the best public health evidence must inform activities in three priority areas to stop this pandemic: (1) coordinated and consistent stay-at-home orders across multiple jurisdictions, including potential nationwide mandates; (2) rapid scale-up of SARS-CoV-2 testing; and (3) improved health care capacity to respond.

## Coordinated Stay-at-Home Orders

There is public health consensus that limiting the number of contacts between persons can slow COVID-19 transmission in a community and give time for health care systems to respond. The most substantial of these approaches is a government order to stay at home except for food and medical needs. Although there are now multiple theoretical and practical models about how stay-at-home orders and travel restrictions could slow COVID-19 transmission, it is clear from all of them that consistency in implementation and communication is key. These policies will only be effective if they are implemented in a coordinated manner across large geographic regions where people commonly move, but there remain multiple examples of these public health interventions not being uniformly implemented. For instance, our city (Atlanta, Georgia) quickly implemented several local variations of stay-at-home recommendations from multiple city and county levels that comprise our metropolitan area, yet the state-wide recommendation was only implemented several weeks later. This meant that people who are told not to come to work in one Atlanta county had no such order where they lived and continued to congregate in public places. This patchwork response is not unique to the United States and illustrates an underlying lack of understanding about how to use these public health measures to slow the transmission of infectious diseases.

This inconsistency in implementing public health measures has also created substantial amounts of public confusion and fodder for social media conspiracy theories, hyperpartisanship, and distrust of experts. COVID-19 is the first true global pandemic of the social media era, offering new opportunities for rapid distribution of accurate public health information to millions of people. Unfortunately, these critical public health communications about actions to take to protect oneself from COVID-19 are not easy to differentiate from inaccurate or even dangerously wrong information. Having correct information that is well reasoned and delivered through consistent messaging are all pillars of behavior change, including changing people’s transmission-related behaviors in response to COVID-19 [9]. Social media is now one of the most predominant ways that people get information, and public health must find better ways to communicate about mitigation plans through these forums.

## Rapid Scale-Up of Testing

Decisions about COVID-19 mitigation policies must be informed by the best epidemiologic information, which requires rapid scale-up of COVID-19 testing. This will require rapid development of new diagnostic tests, laboratory capacity, testing supply chains, and health care personnel to collect the specimens. Novel testing strategies under development, including the use of rapid diagnostic tests, serological tests, and self-collected specimens, will improve our ability to screen a large number of people quickly and give us a new understanding of the extent of exposure, disease, and recovery. This information will be vital to epidemiologic modeling to support information-driven decision making on the appropriate timing and scope of the response. There are also a rapidly growing number of examples of innovative approaches to implementing COVID-19 testing, including some examples of successful large-scale screening programs like drive-up testing in South Korea where thousands of tests were delivered each day [10].

Changing the course of COVID-19 disease in heavily impacted countries such as the United States, will require a massive scale-up of testing compared to what has been conducted to date. For instance, in the United States, the rate of total COVID-19 testing up to this point is just under 3000 tests per 1 million people, or 964,865 overall since January 10, 2020 [11]. That has been an admittedly dismal response to testing, with a focus mainly on those who are most severely ill. This rate of testing does not meet the needs of the health care sector response, much less the needs to better understand COVID-19 epidemiology in a way that will make control measures most effective. We should be testing at least *1 million US residents every week* (0.3% of the population) during this phase of the pandemic. Additionally, there is a need for shorter time from test to results, to better guide care and isolation decisions, and we must find new ways of reaching more people with testing without overburdening our already taxed health care systems.

## Improve the Capacity of Health Care

The control of movement and scale-up of COVID-19 testing will only be successful in truncating the COVID-19 pandemic and reducing lives lost if there is an immediate commitment of resources to improve the capacity of the health care sector to respond. Reports from multiple countries already impacted by COVID-19 predict that health care capacity will be rapidly exceeded as transmission grows under the current predictions of COVID-19 transmission. The ability of the health care sector to respond will certainly require coordination of efforts to increase the capacity of hospital beds, ventilators, protective equipment, and the clinicians who use them.

Protecting the health and safety of health care workers is vital to the health of each of us and to the workings of our health care system. There needs to be a high level of commitment to the safety of health care professionals by providing them with the tools to prevent nosocomial COVID-19 infections. Although this implicitly means making sure all health care workers have appropriate PPEs, this can also come in the form of telemedicine and other virtual care trends such has chatbots that capitalize on advances in technology to provide care for patients outside of a hospital setting until the time hospitalization is needed. This form of care protects our health care workforce and maximizes the scope of care that can be provided with less impact on the hospital setting.

## Conclusion

Our global public health response to COVID-19 will only be successful if we rapidly generate the best data to inform decisions from our political leaders regarding resources and policies to slow transmission and improve our response. This is an unprecedented global public health crisis that will require not only strong political commitment and courage, but also innovation on a capacity and timing scale that was inconceivable 3 months ago. What we do right now and how quickly we do it will directly change how long COVID-19 is with us and how many people will die. It is critical that science-based information guide our public health strategies and that leaders listen to our best information.

Much of public health is about making changes to improve human life but without much announcement. It is impossible to determine the number of lives saved due to epidemiologic research, yet it is unquestionable that our discipline has saved millions of lives, through the implementation of interventions and preventative programs. Our training to understand and use data to protect our communities has not been needed more. It is also our responsibility to use our skills wisely and in a steadfast way that does not bend to the whim of politics, but instead, affirms what we know, loudly, if needed, and highlights what we still need to determine as quickly and accurately as possible to protect our world.

## Abbreviations

COVID-19: coronavirus disease 2019
PPE: personal protective equipment
SARS-CoV-2: severe acute respiratory syndrome-coronavirus 2
WHO: World Health Organization
